# Therapeutic flexible airway endoscopy of small children in a tertiary referral center—11 years’ experience

**DOI:** 10.1371/journal.pone.0183078

**Published:** 2017-08-17

**Authors:** Wen-Jue Soong, Pei-Chen Tsao, Yu-Sheng Lee, Chia-Feng Yang

**Affiliations:** 1 Department of Pediatrics, Taipei Veterans General Hospital, Taipei, Taiwan; 2 Institute of Emergency and Critical Care Medicines, School of Medicine, National Yang-Ming University, Taipei, Taiwan; Boston Children's Hospital / Harvard Medical School, UNITED STATES

## Abstract

**Objectives:**

Use of therapeutic flexible airway endoscopy (TFAE) is very limited in pediatrics. We report our clinical experiences and long term outcomes of TFAE in small children from a single tertiary referral center.

**Methods:**

This is a retrospective cohort study. Small children with their body weight no more than 5.0 kg who had received TFAE between 2005 and 2015 were enrolled. Demographic information and outcomes were reviewed and analyzed from medical charts and TFAE videos.

**Results:**

A total of 313 TFAE were performed in 225 children. The mean age was 3.50 ± 0.24 (0.01–19.2) months old; the mean body weight was 3.52 ± 0.65 (0.57–5.0) kg. A noninvasive ventilation technique, without mask or artificial airway, was applied to support all the procedures. TFAE included laser therapy (39.6%), balloon dilatation plasty (25.6%), tracheal intubation (24.3%) and metallic stent placement (6.4%). Short-length endoscopes of 30–35 cm were used in 96%. All TFAE were successfully completed without serious adverse events or mortality. Mean procedural time was 27.6 ± 16.1 minutes. TFAE resulted in successful extubation immediately in 67.2% (45/67) and 62.8% (118/188) were able to wean off their positive pressure ventilation support in 7 days after procedures. By the end of this study, these TFAE averted the originally suggested airway surgeries in 93.8% (61/65), as benefited from laser therapy, stent implantation, and balloon dilatation plasty.

**Conclusions:**

The TFAE modality of using short-length endoscopes as supported with this noninvasive ventilation and ICU support is a viable, instant and effective management in small children. It has resulted in rapid weaning of respiratory supports and averted more invasive rigid endoscopy or airway surgeries.

## Introduction

Flexible airway endoscopy (FAE) allows direct and dynamic inspection of approachable airways. In pediatrics, thin flexible endoscopes (FE) are available but their use is still restricted for diagnostic FAE (DFAE) [1–5]. Both guidelines of the British Thoracic Society in 2003 [6] and the American Thoracic Society in 2015 [7] have only stated limited applications of therapeutic FAE (TFAE) such as lavage, removal of secretion plugs, expanding collapsed lobes, and aiding endotracheal tube (ET) intubation. These TFAE techniques may not be applicable to very small children because of their narrower airways, poor physiologic reserve, higher sedative risk, and different pulmonary disease entities. The recently reported TFAE [8–11] also rarely discussed the more challenging fields of laser therapy, balloon dilatation plasty (BDP), metallic (balloon expandable) stent implantation and stent plasty. Theoretically, effective TFAE can prevent more invasive interventions of rigid endoscopy (RE) [7] or surgeries such as tracheostomy, laryngotracheal reconstruction, tracheobronchial (TB) plasty, all of which need general anesthesia or even extracorporeal life support (ECLS) [8–13]. Both RE and surgical procedures are technically more complicated and require risky transport to operation room for those small children already cardiopulmonary compromised. Further both services may not be readily accessible or even unavailable. Finally these procedures are mostly expensive and may place financial strain on those resource-limited settings.

For more than two decades, we have gradually developed and employed a convenient and less invasive TFAE modality for our pediatric patients which could undergo immediately following their DFAE. All these procedures used short-length FE of 30–35 cm without inner channel (IC), were performed in side-by-side approach of FAE with the other instruments, and were supported by a novel non-invasive ventilation (NIV) technique [12,13] without ventilation bag, mask or artificial airway, in the pediatric intensive care unit (ICU) setting. Many patients who had been referred from both local and overseas tertiary centers for failed weaning from their prolonged positive pressure ventilation (PPV) could be extubated and avoid open airway surgeries after our TFAE.

The objective of this study is to present our last decade’s experiences of TFAE applications including safety, efficacy and long-term outcomes in a cohort of small children with body weight no more than 5.0 kg.

## Material and methods

### Patient population and data source

This retrospective study involved medical chart and associated-video reviews at our hospital, a university-affiliated tertiary referral teaching hospital. The qualifying repository stored in files and computer which located in our bronchoscopy office. The study period covered an 11-year period from January 2005 to December 2015. Children fulfilling the following criteria were enrolled: 1) They received TFAE procedures within the period from January 2005 to December 2015; 2) Their body weight were no more than 5.0 kg at their first time of TFAE; and 3) Their medical records and TFAE videos could be retrieved for studying.

Data on demographic information, the primary TFAE item (only one of each TFAE counted), procedural success and time, short-term efficacy, adverse effects and long-term outcomes were extracted and analyzed. Procedural success was defined when the TFAE was safely completed. Procedural time was calculated from the beginning to the end of the TFAE as recorded in the videos.

Short-term efficacy was measured based on the success in weaning off respiratory support immediately and on day 7 after TFAE. Our routine weaning protocol is to transition from invasive mode of ET–PPV to non-invasive ventilation via nasal prongs NP–PPV, and finally take off the NP-PPV to spontaneous breathing with oxygen cannula or room air. While maintaining patient’s vital signs and blood gases within acceptable limits, we tolerated permissive hypercarbia and hypoxemia during the weaning process. The accepted blood gas ranges were pH no less than 7.20, PaCO_2_ no more than 70 mmHg and SpO_2_ no less than 85 mmHg.

Long-term outcomes were determined whether the originally proposed airway surgeries from their referring hospitals could be averted for at least one year after their last TFAE (as of the end of 2016). Adverse events during TFAE procedures including significant bradycardia and oxygen desaturation were defined as follows: (1) heart rate less than 100 beats per minute and oxyhemoglobin saturation less than 85% respectively; or (2) each parameter decreased by 10% from their original values for more than one minute. Significant airway bleeding was defined as the requirement of topical cold saline lavage, blood transfusion or beyond. The chest radiograph was checked after each TFAE for any significant new lesions.

Written informed consents, which listed all possible complications and management, of the FAE and associated TFAE were obtained from parents or guardians of the minors before procedures. This study was approved by the Committee for the Protection of Human Subjects in Research of our hospital (IRB-TPEVGH no.: 2016-04-006A) S1 Fig.

### Statistical analysis

Variables were expressed as mean ± standard error or median (range) and were compared using Kruskal–Wallis test. Categorical variables were compared using Chi-square tests. Two-tailed *p*-values <0.05 were considered statistically significant. Data were analyzed using SPSS statistical package, version 19.0 (SPSS, Chicago, IL, USA).

## Results

### Patient characteristics

A total of 313 TFAEs performed in 225 children (103 males) were enrolled (Table 1). Before TFAE, 196 (87.1%) had been staying in ICU for their critical illnesses. 292 (93.3%) TFAEs were executed in the ICU endoscopy room and 21 (6.7%) at ICU bedside. After TFAE, all children were supported and managed in ICU. The mean body weight was 3.52 ± 0.65 Kg. The mean age was 3.50 ± 0.24 months. The smallest and youngest infant was an extreme premature baby of 570 g, when the TFAE aided a correct nasal ET re-intubation immediately after DFAE confirmed a huge iatrogenic vocal cords perforation at 20 minutes of life.

**Table 1:** Therapeutic flexible airway endoscopies in children of body weight no more than 5 kg.

Procedures	Number (%)	Data
Patient / Procedure	225 / 313 (100)	
BW (kg)		
Mean ± SE (median, range)		3.52 ± 0.65 (3.6, 0.57–5.0)
Age (months)		
Mean ± SE (median, range)		3.50 ± 0.24 (4.2, 0.01–19.2)
Procedure items		
Laser therapy	124 (39.6)	
Balloon dilatation plasty	80 (25.6)	
Tracheal intubation	76 (24.3)	
Stent implantation	20 (6.4)	
Forceps management	10 (3.2)	
Tracheobronchial lavage/inflation	3 (0.9)	
Procedural time (minute)		
Mean ± SE (range)		27.6 ± 16.1 (3.0–52.0)

Six items of TFAE procedures were performed with the leading ones including laser therapy (39.6%), BDP (25.6%), tracheal intubation (24.3%), and stent implantation (6.4%). They were all executed immediately after DFAE in the same FAE sessions, except that stent implantations needed extra time to discuss and get consent from parents as well as to prepare appropriate stents. The mean procedural time of these TFAE was 27.6 ± 16.1 (range, 3.0–52.0) minutes.

### Flexible endoscopes and endoscopists

Four types of FE had been used: (a) outer diameter (OD) 2.0 mm, length 60 cm, no IC (inner channel) (LF-P, Olympus); (b) OD 3.2 mm, length 30 cm, no IC (ENF-V2, Olympus); (c) OD 4.8 mm, length 36 cm, IC 2.0 mm (ENF-VT2, Olympus); and (d) OD 3.6 mm, length 60 cm, IC 1.5 mm (FE-2, Olympus). FE with no IC can work alongside with the instruments inside the airway [12, 13]. Except for endotracheal intubation, 96% (240/250) of TFAE used a short-length FE. All TFAE were performed by one of three endoscopists (Soong, Lee and Tsao) who are also well-experienced and qualified neonatologist, pediatric pulmonologist and intensivist in Taiwan.

### Sedations

Procedural sedation of intravenous midazolam (0.3–0.5 mg/kg) and ketamine (0.5–2.0 mg/kg) were recommended. Additional doses of above medications as well as muscle relaxant (succinylcholine) were given as needed to keep patients quiet, motionless or even induced apnea for those prolonged and complicated endoscopy, especially at the critical moment of the therapeutic procedures. Topical airway anesthesia was also applied using 2% lidocaine solution. Vital signs of heart rate, respiration and pulse oximetry were monitored continuously and blood pressures were recorded intermittently throughout the whole course. Nasal cannula, nasal prongs, endotracheal tube and mechanical ventilation were all intentionally removed from patients and replaced with the following NIV management.

### NIV technique

**Fig 1:**
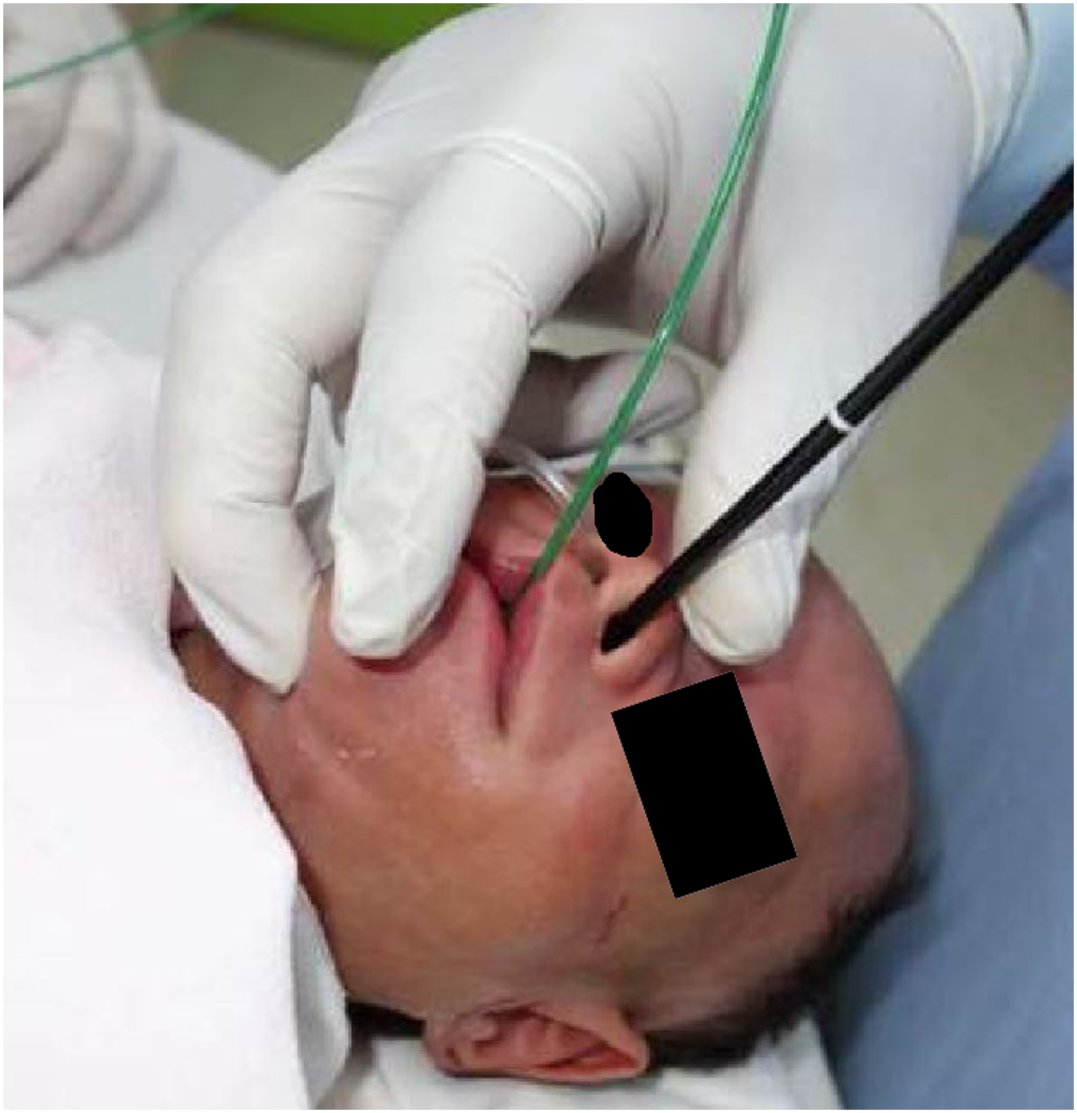
Picture of therapeutic flexible airway endoscopy. A 2.2 kg small premature infant undergoing therapeutic flexible airway endoscopy, a short-length 3.2 mm OD endoscope via left nose, a 6F oxygen catheter via right nose and a 5Fr. balloon catheter via mouth. Endoscopist’s right hand puts around nose and mouth for proceeding nose and mouth-closure.

The NIV technique by means of nasopharyngeal oxygen with nose-closure and abdomen-compression (PhO_2_-NC-AC) [12], was applied intermittently to all these FAE (Fig 1). It started from the preparatory period, throughout the whole procedure, and might last for further three minutes after finishing FAE as compensation if indicated. The rhythm and intensity in performing this NIV were adjusted during FAE procedures, depending on patient’s heart beat and oxygen saturation on the monitor. This NIV had been also used concurrently with the ECLS in 4 children undergoing stent placement for their failed extubation due to persistent and severe tracheobronchomalacia after surgery. Additional advantage of using this NIV was the optional expansion of both upper and low airway lumens, as controlled by endoscopist, for better assessment of airway condition while doing TFAE.

After procedure, NP-PPV was routinely supported to maintain physiology within the acceptable limits (as previous descripted) and then gradually weaned off depending on the respiratory status. All patients received short course of inhaled budesonide, intravenous antibiotics and anti-emetics for 3 to 7 days. Follow-up FAE was performed 3 to 5 days later to assess the results if indicated.

### Four leading TFAE procedures

#### Laser therapy

**Fig 2:**
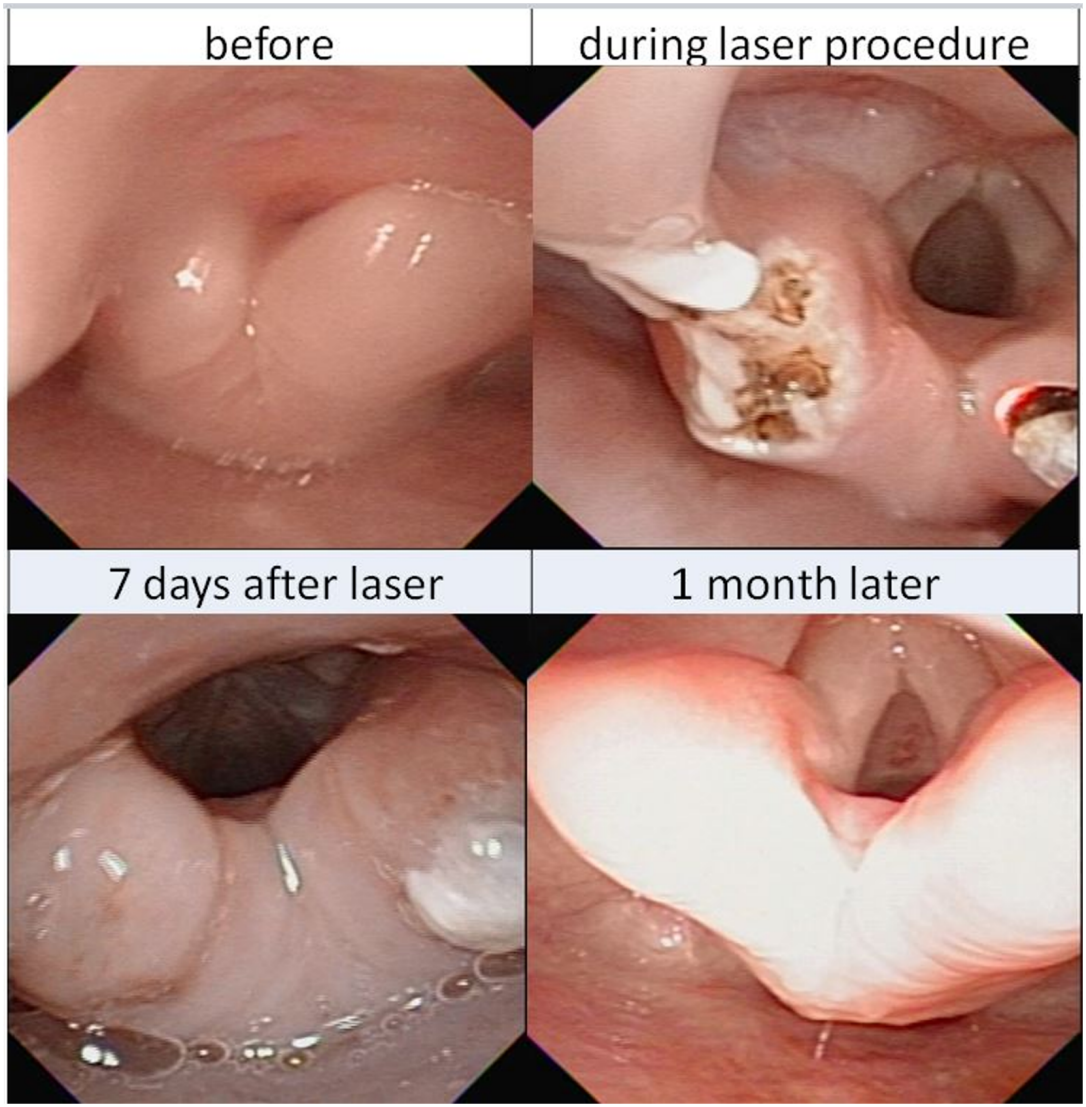
Sequential images of laser supralaryngoplasty. Before, during and after the laser ablation of the redundant tissues over bilateral arytenoids with flexible airway endoscopy in severe laryngomalacia. One month later, it became normal.

**Fig 3:**
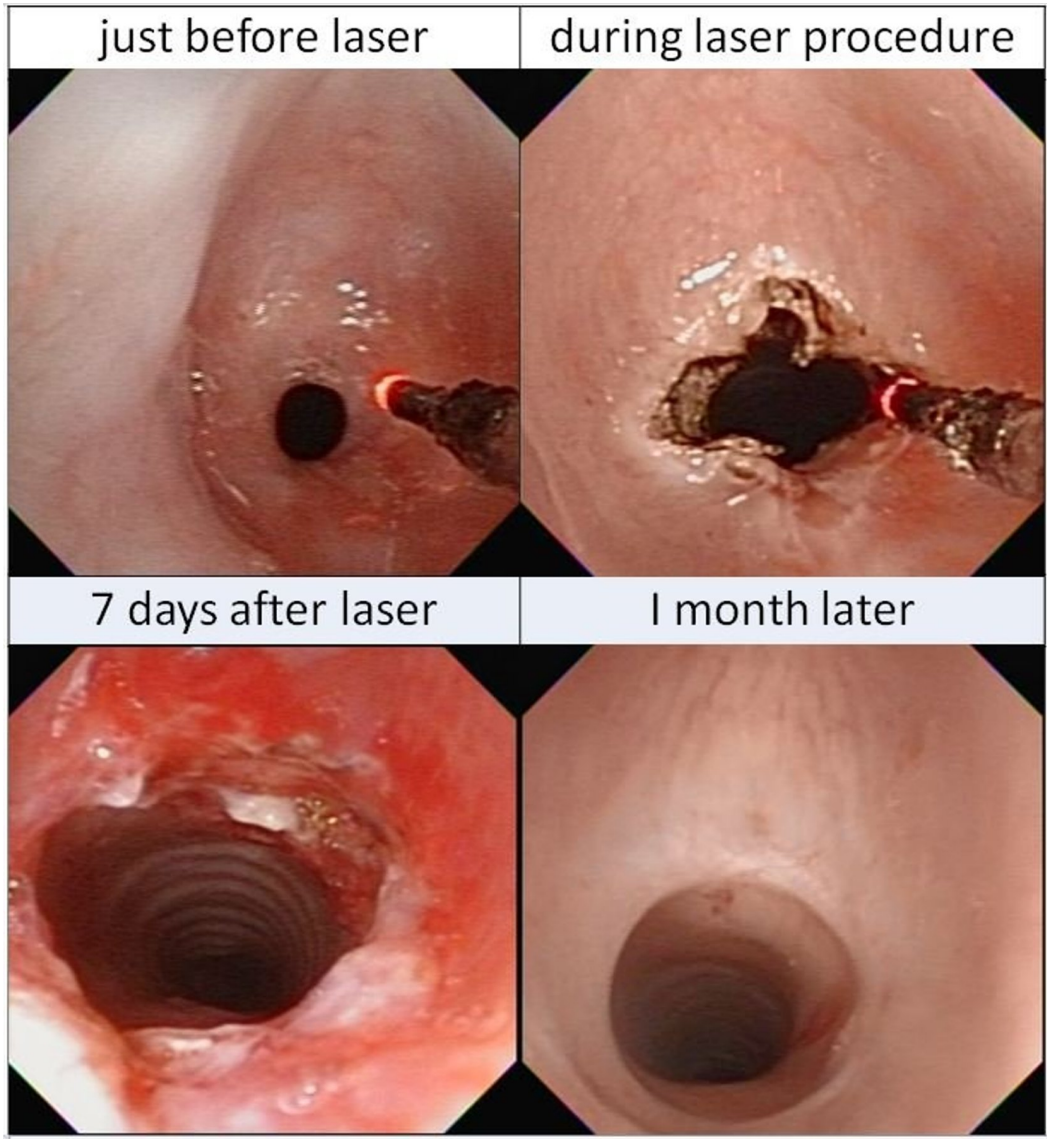
Sequential images of laser therapy in subglottic stenosis. Before, during and after the laser ablation with flexible airway endoscopy on a severe subglottic stenosis (<2 mm). One month later, it became almost normal.

A total of 124 laser procedures were conducted in 101 children (Table 2) for upper (Fig 2) and lower (Fig 3) airway lesions. Among them, 115 (92.7%) were done with a short length FE of OD 4.8 mm (ENF-VT2) but another 9 (7.3%) with a longer FE of OD 3.6 mm (FE-2) for passing via narrow TB lumens. Laser supraglottoplasty for severe laryngomalacia [14] was the leading procedure, which was performed in DTFE confirmed severe laryngomalacia children. The laser fiber passed through the IC of FE. The mean procedural time was 22.7 ± 9.3 minutes.

**Table 2:** Laser management of therapeutic flexible airway endoscopy in children of body weight no more than 5 kg.

Laser therapy	Number (%)	Suggested surgery^a^
Infant / Procedure	101 / 124 (100)	38 / 38
Indications		
Laryngomalacia	81 (65.3)	15^a^
Subglottic stenosis	21 (16.9)	11
Tracheobronchial lesions^b^	12 (9.7)	6
Vallecular cyst	6 (4.8)	3
Subglottic hemangioma	4 (3.2)	3
Procedural time (min)		
Mean ± SE; range	22.7 ± 9.3; 10–42	

^a^number of suggested surgery from the referring hospitals.

^b^tracheostomy, stenosis or granulation.

Before procedures, 96 children had received PPV support (14 with ET and 82 with NP), and all their respiratory supports were removed prior to these laser therapies. Seven days after laser treatment, only 29 children (2 with ET and 27 with NP) were still PPV- dependent. Originally, thirty-eight surgeries were considered from referring hospitals upon admission. Finally, only 4 children with refractory subglottic stenosis failed to the laser therapy and received tracheostomy. The remaining thirty-four surgeries were successfully averted.

### Balloon dilatation plasty

**Fig 4:**
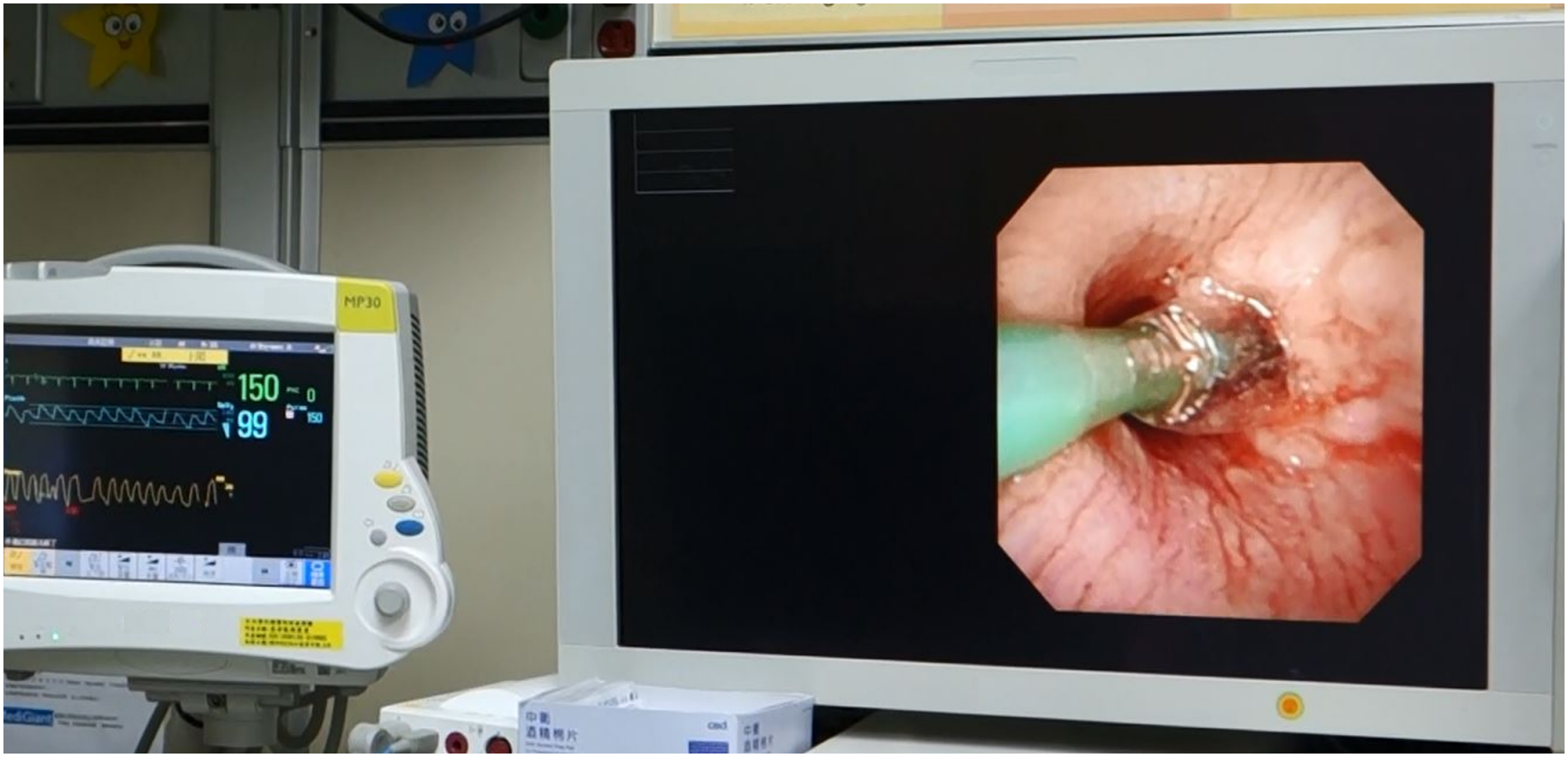
Picture of balloon dilatation plasty. During the therapeutic flexible airway endoscopy of balloon dilatation plasty in right main bronchus. The synchronized monitor shows spontaneous breathing and stable vital condition.

Eighty BDP were performed in 55 children (Table 3). The FAE worked as a visual guide with an angioplasty balloon catheter (Boston Scientific, Mustang) of appropriate dimension placed alongside in the target TB lumens (Fig 4) [12]. The balloon catheter was stiffed with a guide wire inside for easy manipulation [15]. Thirty-eight BDP corrected lumen narrowing which included 21 tracheal, 9 bronchial, 5 nasal tract and 3 choanal atresia lesions. Twenty-four BDP were used for expansion and estimation of the dimension of target TB lumens, an essential step prior to stent placement. Ten stent plasty were performed for the repair and re-expansion of distorted and loose stents. Five BDP were done to compress the granulomas and restore adequate lumen patency. Three BDP assisted the stent retrieval, This required inserting a deflated balloon catheter underneath the target stent, then inflating the balloon to detach the stent from the underlying airway mucosa, and finally grasping the loosened stent with forceps via RE.

**Table 3:** Balloon dilatation plasty of therapeutic flexible airway endoscopy in children of body weight no more than 5 kg.

Balloon dilatation plasty	Number (%)	Suggested surgery^a^
Infant / Procedure	55 / 80 (100)	12 / 12
Indications		
Correct lumen narrowing	38 (47.5)	10
Pre-stent lumen measurement	24 (30.0)	
Stent plasty^b^	10 (12.5)	
Crush granuloma	5 (6.3)	2
Destroy stent for retrieval^c^	3 (3.8)	
Procedural time (minute)		
Mean ± SE; range	24.7 ± 12.3; 12.0–45.0	

^a^number of suggested surgery from the referring hospitals.

^b^repair damaged or distorted stent.

^c^detach stent for easy retrieval by forceps with rigid scopy.

Thirty ET-PPV were intentionally removed prior to BDP. After procedures, 17 children succeeded in immediate extubation whereas another 22 children could have their ET removed by 7 days later. The mean procedural time was 24.7 ± 12.3 minutes. Among them, all 12 surgeries initially suggested were successfully averted.

### Tracheobronchial intubation

Seventy-six endoscopy-aided ET intubations were performed in 44 children (Table 4). All were done through the nasal tract route except four in trans-oral approach. Both the upper and low airway problems were encountered. Thus more sophisticated endoscopic manipulation was required inside the distorted and obstructed airways than traditional simple FE-aided intubation. Among them, the most common cause of difficult intubation came from upper airway pathology in 24 (31.6%) cases including Pierre-Robin complex, huge tongue mass, laryngeal cleft, laryngeal inlet mass, iatrogenic vocal cords perforation, and subglottic perforation. Thirty-five (46.1%) intubations were performed in unordinary indications. These included 20 TFAE which guided the correct positioning of the end and/or the side holes of the endotracheal tube to prevent lumen blockade or injury to existing tracheal lesions during dynamic breathing movements [16]. Eight selective bronchial intubations were conducted to achieve one-lung ventilation to manage 5 cases of pulmonary bleeding, 2 persistent bronchopleural fistulas and 1 child with huge lobar pneumatocele. Seven aided intubations were done for children with pre-existing tracheal stents who required intubation for other management. This endoscopic guided approach prevented the damage of existing stents by blind intubation. The mean procedural time was 4.8 ± 2.5 minutes.

**Table 4:** Tracheobronchial intubation of therapeutic flexible airway endoscopy in children of body weight no more than 5 kg.

Scopy-aided intubation	Number (%)
Infant / Procedure	44 / 76 (100)
Indications	
Difficult upper airway	24 (31.6)
Tracheal lesions^a^	20 (26.3)
High intracranial pressure	12 (15.8)
Selective bronchial intubation	8 (10.5)
Presence of tracheal stent	7 (9.2)
Respiratory failure	5 (6.6)
Procedural time (minute)	
Mean ± SE; range	4.8 ± 2.5; 2.0–9.5

^a^include 5 stenosis, 5 malacia, 4 granuloma, 3 perforation and 3 surgical wounds.

### Stent implantation

**Fig 5:**
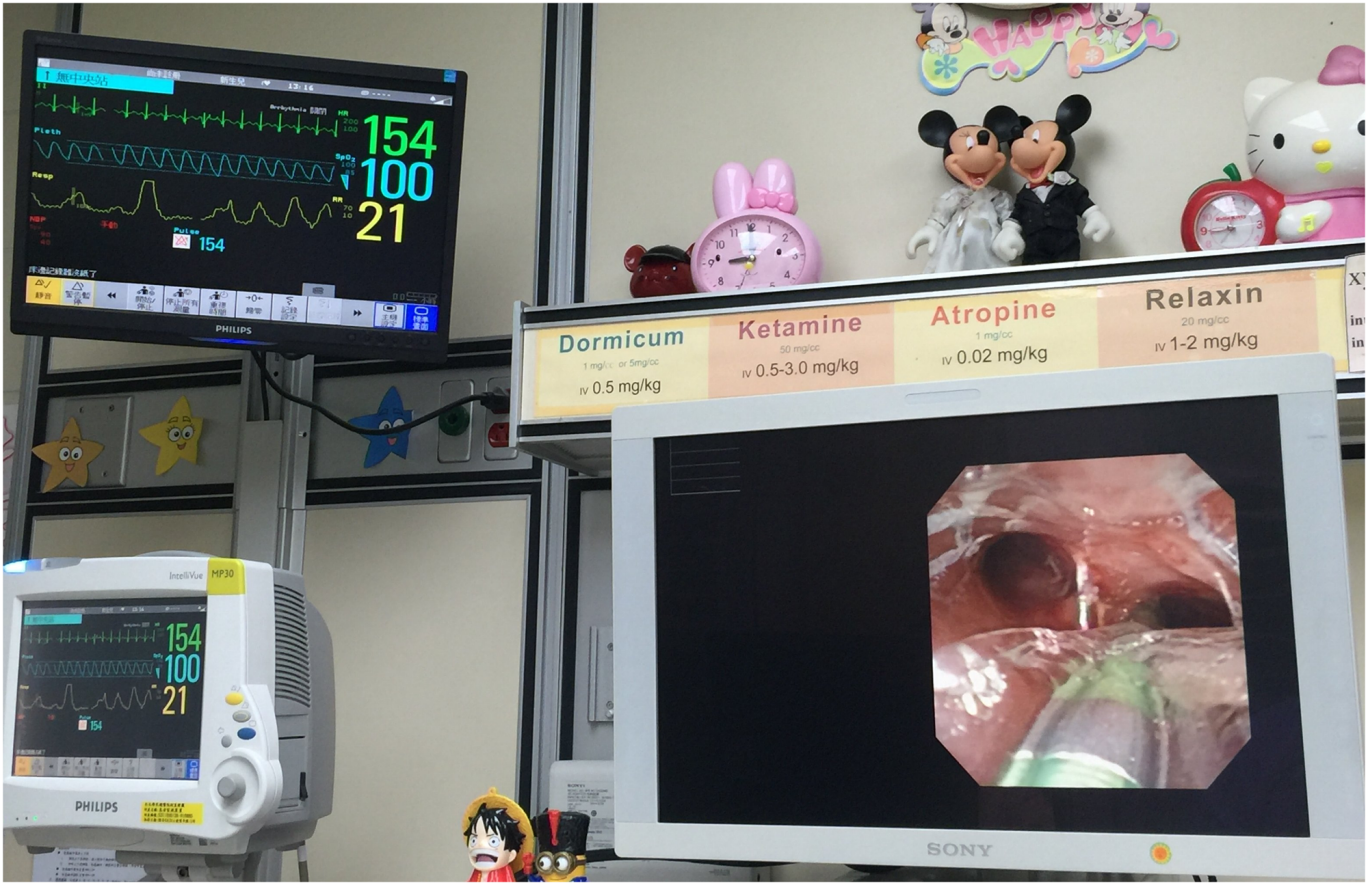
Picture of carina stent implantation. During the procedure of left carina stent implantation, a deflating balloon inside the stent lumen after creating a right carina hole. The synchronized monitor shows spontaneous breathing and stable vital signs.

Twenty stents were implanted in 15 children (Table 5). The indication was severe TB narrowing (stenosis or malacia) resulting in prolonged ET intubation and PPV dependence. BDP or laser therapy of TFAE did not improve their condition and the patients still suffered from frequent life-threatening episodes. Stent placement was considered as the last option before proceeding to more invasive surgical interventions. Four stents were placed after sliding tracheoplasty due to persistent lumen collapse (as mentioned above). Stents were of metallic mesh type (IntraTherapeutics Inc, MN, USA) which could be further expanded by technique of BDP to accommodate the growing TB lumens. These placements and subsequent BDP were all performed with a short-length FE of OD 3.2 mm. The smallest body weight was 2.3 kg and the youngest infant was 10 days old. Three carinal stents were placed in 3 growing extreme premature children on prolonged invasive ventilation for the correction of their peri-carinal malacia. These stents span from one main bronchus distally and lower tracheal region proximally, which cover the lesions around the peri-carinal region. A big side-hole was then created in the stents by BDP adjacent to the contralateral bronchial orifice (Fig 5). The mean procedural time was 34.4 ± 13.3 minutes. Fourteen ET-PPV were purposely removed for doing these procedures and no children required re-intubation or subsequent tracheobronchial surgeries afterward. Only that one infant with pulmonary sling underwent the vascular correction 3 years later.

**Table 5:** Tracheobronchial stent Implantation of therapeutic flexible airway endoscopy in children of body weight no more than 5 Kg.

Stent implantation		Number (%)	Data
Infant / Stent		15 / 20 (100)	
Age (month)	Mean ± SE		5.8 ± 2.9
	Median; range		5.3; 0.3–10.4
Body weight (Kg)	Mean ± SE		3.9 ± 0.7
	Median; range		4.0; 2.3–5.0
Locations	Trachea	10 (58.3)	
	Carina^a^	3 (12.5)	
	Bronchus	7^b^ (29.2)	
Procedural time (minute)	Mean ± SE		34.4 ± 13.3
	Median; range		35.5; 20.0–55.4
Stented period (month)			
Expired stent		5 / 6 (30.0)	
	Mean ± SE		18.3 ± 9.3
	Median; range		21.6; 1.4–36.5
Retrieved stent		2 / 3 (15.0)	
	Mean ± SE		48.6 ± 36.7
	Median; range		40.2; 16.7–87.4
Remained stent		8 / 11 (55.0)	
	Mean ± SE		62.2 ± 31.7
	Median; range		55.6; 29.2–120.8

^a^one stent placed from main bronchus cross over carina and end at the low trachea.

^b^2 in right main bronchus and 5 in left main bronchus.

At the end of this study, 5 patients with 6 stents were expired due to their underlying severe cardiac and brain problems. The mean period of their stents was 18.3 ± 9.3 months. In the 10 surviving patients, 3 stents in 2 patients were completely removed after the mean period of 48.6 ± 36.7 months. By the end of this study, 11 stents still remained in 8 patients with the mean period of 62.2 ± 31.7 months.

### Others

Ten forceps procedures included retrieval of 6 iatrogenic TB foreign bodies (2 foil papers [15], 2 fractured suction tubes in bronchi and 2 broken stent segments) and removal of 4 tracheal granulomas. Three bronchoalveolar lavage followed by gas inflation for pulmonary collapse succeeded in lung re-expansion, as confirmed upon follow-up chest radiographs.

### Adverse events

During these 313 TFAE procedures, all episodes of bradycardia and desaturation were transient which could be rapidly corrected and restored to acceptable levels (≧100 beats/min and ≧90%, respectively) within 60 seconds by using our novel NIV maneuver. No any artificial airway or other types of PPV needed to be applied. After TFAE, no unplanned ET intubation, PPV, or intravenous adrenergic medications were required. All TFAE associated airway bleeding was mild and self-limiting. Two small right pneumothoraces were noted, which resolved on conservative management without needle aspiration or tube drainage. No TFAE-associated mortality was recorded.

### Short-term efficacy of weaning respiratory supports

Except for scope-aided intubation, TFAE, can result in early weaning of respiratory support (*p* < 0.05) as shown in Table 6. Before TFAE, there were 67 ET-PPV supports, which immediately decreased to 22 with 45 transitioned to NP-PPV after TFAE, and further down to 11 in 7 days. Similarly, 121 children initially required NP-PPV support before TFAE but only 59 remained NP-PPV dependent within 7 days. Sixty two children only required oxygen cannula or simply room air afterward. To sum up in a total of 188 PPV before TFAE, 118 (62.8%) were successfully weaned within 7 days after TFAE. The success of weaning off PPV was mostly attributed to the three items of TFAE, including laser therapy (67, 69.8%), BDP (28, 47.5%) and stent implantation (15, 75.0%). Finally, all survivals were able to wean off to spontaneous breathing in room air.

**Table 6:** Weaning of respiratory supports after therapeutic flexible airway endoscopy in children of body weight no more than 5.0 kg.

Procedures	Number	Before / immediate after / within 7 days		
		ET-PPV	NP-PPV	O_2_ cannula or RA
Laser therapy	124	14 / 5^a^ / 2^a^	82 / 115^a^ / 27^a^	28 / 4^a^ / 95^a^
Balloon dilatation plasty	80	30 / 13^a^ / 8^a^	29 / 41^a^ / 23	21 / 26 / 49^a^
Stent implantation	20	14 / 0^a^ / 0^a^	6 / 20^a^ / 5	0 / 0 / 15^a^
Foreign body retrieval	10	6 / 2 / 0^a^	4/ 8 /2	0 / 0 / 8^a^
TBL and inflation	3	3 / 2 / 1	0 / 1 / 2	0 / 0 / 0
Total	237	67 / 22^a^ / 11^a^	/ 185^a^ / 59^a^	49 / 30^a^ / 167^a^

^a^*p* <0.05; ET, endotracheal tube; PPV, positive pressure ventilation; O_2_, oxygen; RA, room air.

### Long-term prognosis of averting surgery

Initially, 65 surgical interventions had been suggested in the “case review conference” of the referring hospitals because of failed conservative treatment (Table 7). Ultimately, at the end of this study, only 4 tracheostomies were done for refractory subglottic stenosis and one patient underwent the correction of pulmonary sling 3 years later. Eventually, 61 (93.8%) airway surgeries had successfully been averted. They were all benefited from the three TFAE procedure items of laser therapy, stent implantation and BDP.

**Table 7:** Avert surgical interventions by therapeutic flexible airway endoscopy (TFAE) in children of body weight no more than 5.0 kg.

TFAE procedures	TFAE number	Suggested surgery^a^ (100%)	Avert Surgical Interventions		
			TS / LTR	TBP + ECLS	Subtotal (%)
Laser therapy	124	38	29	5	34 (89.5)
Stent implantation	20	15	8	7	15 (100)
Balloon dilatation plasty	80	12	8	4	12 (100)
Total	224	65	45	16	61 (93.8)

/, with or without; ECLS, extracorporeal life support; LTR, laryngotracheal reconstruction; TS, tracheostomy; TBP, tracheobronchoplasty.

^a^number of original suggested from the referring hospitals.

## Discussion

To the best of our knowledge, this is the most extensive report covering the largest case number of TFAE done in small children. Many unique TFAE applications such as laser therapy, BDP, stent implantation and plasty have rarely been mentioned before [6–11]. Additionally, it also highlights that these TFAE techniques can be safely, timely and effectively executed by well-experienced endoscopists without using any ventilation bag, mask, airway, or mechanical ventilation in this high-risk population.

Traditionally, except for ET intubation, TB lavage and stent plasty, most of the aforementioned therapies may be carried out with rigid endoscopy or surgical procedures with expected results. However the latter two more invasive interventions require arduous measures such as transportation, operation room service, general anesthesia, endotracheal intubation and mechanical ventilation, or even ECLS, all of these interventions are more complicated and risky in these very small sick children. Further, a rigid instrument such as the RE may itself distort and damage their fragile upper and low airway lumens, and is inappropriate or incapable of managing stent associated problems and lesions [14–21]. Though the facilities and capabilities of both RE and surgery are available in our center, these are not our priorities of management. Instead, FAE is better for its readily availability and simplicity with lesser airway distortion and injury, as well as providing better dynamic inspection of airway condition. DFAE has already been recognized as an essential tool for early and accurate detection of airway lesions. Further, after appropriate training, TFAE can also be another very effective and practical technique. As presented here, all these TFAE were safely performed immediately after the corresponding DFAE. This combined DFAE and TFAE in the same session has actually decreased waiting time, medical expense, and stress on both physicians and patients on one hand, and avoided more invasive interventions and their associated iatrogenic damages on the other hand. These benefits have already been demonstrated in the previous reports [12–21].

One of our main concerns of TFAE in small children is whether the FE caliber is small enough to fit the limited airway lumens. In this study, we have proved that an OD 3.2 mm FE could smoothly work together with another instruments alongside within the target lumens. Theoretically, more adverse effects can occur during TFAE than DFAE because of requirement of more sedation, and manipulation, as well as more complicated and prolonged procedural times. Effective and skillful techniques of TFAE, constant vigilance on anticipated risk as well as more liberal use of our novel NIV during all procedures are therefore necessitated. A short-length FE of 30–35 cm is easier to handle than the traditional long one of 60 cm and is adequate to reach the target TB lumen in our small children.

The most crucial element leading to success in performing TFAE is to ensure endoscopist competency and patient safety. Capability in maintaining adequate oxygenation, ventilation, circulation, and keeping clear visual field endoscopically are essential, especially around the time when patients are under heavy sedation, or even drug-induced apnea prior to stent implantation. This NIV technique [12] can easily and optionally provide both inspiration and expiration by simply doing nose-closure and abdomen-compression, respectively, in the presence of a small pharyngeal oxygen catheter in site. It surpasses the traditional PPV support as this NIV causes less impediment and distortion, and does not share the airway with other space-occupying devices such as ventilation bag, facemask, ET, ventilator, or RE. Therefore, it can offer more operative space over the patient's face, entrances of nose and mouth and intra-airway lumens for easier manipulation of TFAE and interventional instruments. The endoscopist himself can optionally control the rhythm and intensity of this PPV to eliminate hypoxia, desaturation and simultaneously get more dynamic and comprehensive inspection in the target lumens. Therefore, using this NIV can provide a significant yield in both DFAE and TFAE. Our previous piglet [20] and clinical [12] studies have already demonstrated that it could effectively support and rapidly correct hypoxia, hypercapnia and bradycardia, even during very complicated DFAE procedures. Healthcare professionals who have become familiar and skillful in performing this life-supporting and rescuing NIV may reap the benefits and be more confidence in their medical practice.

Currently, the majority of FAE in children are performed by pediatric pulmonologists or otolaryngologists who may not be familiar in management of small and sick children. With the high occurrence of respiratory pathologies in this population, we suggest that it may be necessary for pediatric bronchoscopists and assistants to receive proper training in the basic neonatal and ICU care as well as TFAE techniques. This strategy has also been recommended by Kohelet et al [22].

Several limitations are present in this study. As a retrospective study, there is no control group because we have routinely and effectively performed these TFAE modalities for more than 15 years. There were no records reporting in detail the frequency and severity of the adverse events. Theoretically, children of younger age and smaller body weight may have more adverse events and worse outcomes. However, the occurrence of these complications are highly influenced by the underlying disease severity, and the variations while executing this NIV such as frequency, time and tightness of nose-closure and depth of abdomen-compression, all of which will inevitably affect the effectiveness of this NIV support. In this study, both hypoxia and desaturation were not significant and could be rapidly corrected without abolishing the TFAE. Further prospective study on this field may be required to standardize the execution of this NIV. Limited space in this text does not allow further analysis and discussion of these TFAE techniques in detail as against sub-groups of different age and body weight. Further, we have not discussed the use of newer tiny FE 2.8 mm OD with 1.2 mm IC (BF-XP60 OES, Olympus) as it was still not available during the studied period.

### Conclusion

This report suggests that this TFAE modality of using short-length FE coupled with this NIV and ICU support is safe, viable, timely and effective management that can avert more invasive RE or open surgical interventions in small sick children. Further this report has stated the benefits of therapeutic flexible airway endoscopy, which can definitely encourage further training and advancement in this field.

## Supporting information

S1 Fig Clinical Trial/Research Approval Letter: IRB-TPEVGH No.:2016-04-006A. (PDF)
