# Supplementary material for: Therapeutic flexible airway endoscopy of small children in a tertiary referral center—11 years’ experience
Source: PLoS One. 2017 Aug 17;12(8):e0183078. doi: 10.1371/journal.pone.0183078 (PMC5560590; doi:10.1371/journal.pone.0183078)
Supplement: S1 Fig — (PDF) [file pone.0183078.s001.pdf]

臺 北 榮 民 總 醫 院
TAIPEI VETERANS GENERAL HOSPITAL
201 SHIH-PAI ROAD, SEC. 2
TAIPEI, TAIWAN 11217
REPUBLIC OF CHINA
TEL: (886)-2-2871-2121

Clinical Trial/Research Approval Letter

May. 6, 2016

IRB-TPEVGH No.: 2016-04-006A
Protocol Title: Therapeutic Flexible Airway Endoscopy in Critically Ill Infants in a Tertiary Referral Center
Department/Principal Investigator: Department of Pediatrics/ Soong, Wen-Jue
Version date of documents:

1. Protocol : Version 1./ Date :20160301
2. Chinese/English Synopsis: Version 1./ Date :20160301
3. Informed Consent Form: Waived
4. DSMP: Version 1./ Date :20160301

According to the written operating procedures, GCP, and the applicable regulatory requirements, this study project is approved by (the 76th meeting of) the Institutional Review Board (1) of Taipei Veterans General Hospital on Apr 11, 2016. This approval is valid for 1 year till Apr 10, 2017.
The board is organized under, and operates according to International Conference on Harmonisation (ICH) / WHO Good Clinical Practice (GCP) and the applicable laws and regulations.
The principal investigator is required to report Serious Adverse Events and Unanticipated Problems in accordance with the governmental laws and regulations and TPEVGH requirements.
The principal investigator is required to submit the application for extension at least 6 weeks before the expiration date. (If indicated by the regulations and laws, this project should be taken after the approval of Ministry of Health and Welfare, R.O.C.)

臺北榮民總醫院 人體試驗委員會 Institutional Review Board Taipei Veterans General Hospital

Shung-Tai Ho

Shung-Tai Ho, M.D.
Chairman
Institutional Review Board
Taipei Veterans General Hospital
Taiwan, R.O.C.
